# A nested PCR-based point-of-care multiplex test for detection of bacterial pathogens in cerebrospinal fluids

**DOI:** 10.3389/fcimb.2026.1781397

**Published:** 2026-04-14

**Authors:** Jianing Wu, Zijin Zhao, Pai Pang, Yanqing Tie, Shihao Jiao, Duoxiao Zhang, Xingyu Liu, Jie Wang, Yuxin Wang, He Tan, Mengchuan Zhao, Pei Zhao, Juntao Meng, Zhiqiang Han, Shijue Gao, Xinxin Shen, Xuejun Ma, Zhishan Feng

**Affiliations:** 1 Graduate School, Hebei North University, Zhangjiakou, Hebei, China; 2 Department of Clinical Laboratory, Hebei General Hospital, Shijiazhuang, Hebei, China; 3 National Key Laboratory of Intelligent Tracking and Forecasting for Infectious Diseases, NHC Key Laboratory of Medical Virology and Viral Diseases, National Institute for Viral Disease Control and Prevention, Chinese Center for Disease Control and Prevention, Beijing, China; 4 Hebei Key Laboratory of Molecular Medicine, Shijiazhuang, Hebei, China; 5 Hebei Clinical Research Center for Laboratory Medicine, Shijiazhuang, Hebei, China; 6 Graduate School, North China University of Science and Technology, Tangshan, Hebei, China; 7 Graduate School, Hebei Medical University, Shijiazhuang, Hebei, China

**Keywords:** cerebrospinal fluid, multiplex detection, multiplex one-tube nested real-time polymerase chain reaction (mOTN-PCR), point-of-caretesting, point-of-care testing, *Streptococcus pneumoniae*, *Streptococcus agalactiae*, *Streptococcus pyogenes*

## Abstract

**Objective:**

To integrate a multiplex one-tube nested real-time polymerase chain reaction (mOTN-PCR) with a point-of-care testing (POCT) instrument for rapid, sensitive detection of *Streptococcus pneumoniae* (SPN), *Streptococcus agalactiae* (GBS), and *Streptococcus pyogenes* (GAS) in cerebrospinal fluid (CSF), achieving an automated “sample-in, result-out” workflow.

**Method:**

The sensitivity of the mOTN-PCR was assessed using recombinant plasmids, and its specificity was evaluated using common pathogens associated with CSF infections. Simulated CSF samples were prepared using both manual nucleic acid extraction and fully automated nucleic acid extraction (POCT instrument), and then detected in parallel by mOTN-PCR and conventional real-time PCR. The clinical performance of the mOTN-PCR-POCT assay was further evaluated using 109 clinical samples (36 clinical CSF samples and 73 non-CSF clinical samples), and compared with the manual nucleic acid extraction followed by the conventional real-time PCR method.

**Results:**

The mOTN-PCR achieved detection limits (LOD) of 5, 10, and 10 copies/μL for SPN, GBS, and GAS plasmids, respectively, with no cross-reactivity. In simulated CSF samples, the LODs were 20, 10, and 20 CFU/mL for SPN, GBS, and GAS, respectively, using manual extraction, within 4 hours. The mOTN-PCR-POCT method consistently detected all three pathogens at 20 CFU/mL, reducing total testing time to approximately 2 hours. Clinical sample testing revealed that the mOTN-PCR-POCT identified three additional positives in samples previously deemed negative by conventional real-time PCR, demonstrating superior detection capability and a more streamlined operational workflow of the mOTN-PCR-POCT.

**Conclusion:**

The mOTN-PCR-POCT was demonstrated to be simple to operate, requires no culture, and enables rapid, highly sensitive detection of SPN, GBS, and GAS in CSF.

## 1 Introduction

Central nervous system (CNS) infections, particularly acute bacterial meningitis, represent a critical illness characterized by rapid onset, swift progression, and extremely high rates of mortality and disability, posing a significant threat to global public health (van de Beek et al., 2004; Jian et al., 2025). Statistics indicate that this disease causes approximately 318,000 deaths annually, with its burden being particularly pronounced in low- and middle-income countries and regions experiencing large-scale devastating epidemics (Hasbun, 2022). Although vaccination has significantly reduced its incidence, the latest global meta-analysis indicates that the disease burden of bacterial meningitis remains substantial, with an overall case-fatality rate still as high as approximately 18% (Van Ettekoven et al., 2024). Among various pathogens, *Streptococcus pneumoniae* (SPN), *Streptococcus agalactiae* (GBS), and *Streptococcus pyogenes* (GAS) are the primary causative agents of bacterial meningitis. SPN is the most common cause of bacterial meningitis worldwide, accounting for up to 72% of cases in individuals aged 16 years and older, with a case fatality rate of approximately 24% (van de Beek et al., 2021; Hasbun, 2022; Van Ettekoven et al., 2024). Notably, SPN is also one of the pathogens prone to false-positive results in molecular diagnostics, which undoubtedly increases the difficulty of its accurate detection (Wagner et al., 2018). GBS is the primary causative agent of bacterial meningitis in newborns and infants (van de Beek et al., 2021). The disease has an acute onset, often occurring within the first week after birth, and frequently progresses to systemic infection, with a mortality rate reaching 16% (Van Ettekoven et al., 2024). Compared to SPN and GBS, meningitis caused by GAS is relatively uncommon but often presents with a fulminant course, carrying a mortality rate as high as 20%. Approximately 33% of survivors experience residual neurological sequelae, posing a significant threat to human health (Marquardt et al., 2024).

Currently, laboratory diagnosis of bacterial pathogens in cerebrospinal fluid (CSF) primarily relies on traditional microbiological methods, including Gram staining and CSF culture. Among these, Gram staining is frequently used as a preliminary screening method for detecting bacteria in CSF due to its rapidity and simplicity. Although this method exhibits extremely high specificity, its sensitivity varies significantly among different pathogens, and results are easily influenced by prior antibiotic use (van de Beek et al., 2004; Pizon et al., 2006; Fitch and van de Beek, 2007). CSF culture has long been regarded as the “gold standard” for diagnosis, yet its application faces significant limitations: extended detection time (typically 24–72 hours), susceptibility to various confounding factors, and reduced positivity rates following antibiotic treatment (Brouwer et al., 2010; Michael et al., 2010; Van Dong et al., 2025). These limitations may lead to delayed or missed clinical diagnoses, thereby missing the optimal window for intervention and consequently affecting patient prognosis. Consequently, reliance on traditional culture methods fails to meet the urgent need for early diagnosis, highlighting the necessity of developing non-culture-based, rapid, and accurate novel detection technologies. Advances in molecular diagnostics offer new solutions for rapid pathogen identification in CNS infections. Currently, molecular detection methods widely adopted in clinical practice primarily include PCR and its derivative technologies, such as real-time PCR, along with various isothermal amplification techniques (Schuurman et al., 2004; Parida et al., 2006; Welinder-Olsson et al., 2007; Nagdev et al., 2011; Sun et al., 2023). Although these methods have shortened diagnostic time to some extent, their sensitivity remains inadequate for CSF samples with extremely low pathogen loads or samples that have undergone antimicrobial therapy, posing a high risk of false negatives (Olie et al., 2024). Therefore, there is an urgent need to develop new CSF pathogen detection methods that are simpler to operate, faster, and more sensitive to enhance early diagnosis and improve patient prognosis.

Nested PCR significantly enhances detection sensitivity through two sequential amplification reactions. However, traditional two-step nested PCR requires decapping and dilution of the first-round amplification products to serve as templates for the second round, a procedure that substantially increases the risk of aerosol contamination and compromises the reliability. To enhance detection sensitivity and specificity while reducing operational steps and contamination risks, our research group previously developed a novel one-step, single-tube nested PCR method based on locked nucleic acid (LNA)-modified outer primers and has been successfully applied to detect various viral and bacterial pathogens (Zhao et al., 2019 , Zhao et al., 2020; Zhang et al., 2020). This one-step nested PCR eliminates lid-opening steps, effectively preventing contamination, and demonstrates higher sensitivity and specificity than conventional real-time PCR. However, it still faces limitations such as extended detection cycles, complex LNA-modified primer design, and high synthesis costs (typically several times higher than standard primers).

To address these limitations, this study aims to develop a novel POCT assay by integrating a multiplex one-tube nested real-time polymerase chain reaction (mOTN-PCR) with a point-of-care testing (POCT) instrument. This approach establishes a fully automated, closed-system nucleic acid extraction and detection suitable for clinical point-of-care use. This approach enables rapid, highly sensitive, and specific detection of SPN, GBS, and GAS in CSF. Its clinical performance was evaluated through comparison with conventional real-time PCR.

## 2 Materials and methods

### 2.1 Sample and strain sources and nucleic acid extraction

**Figure 1:**
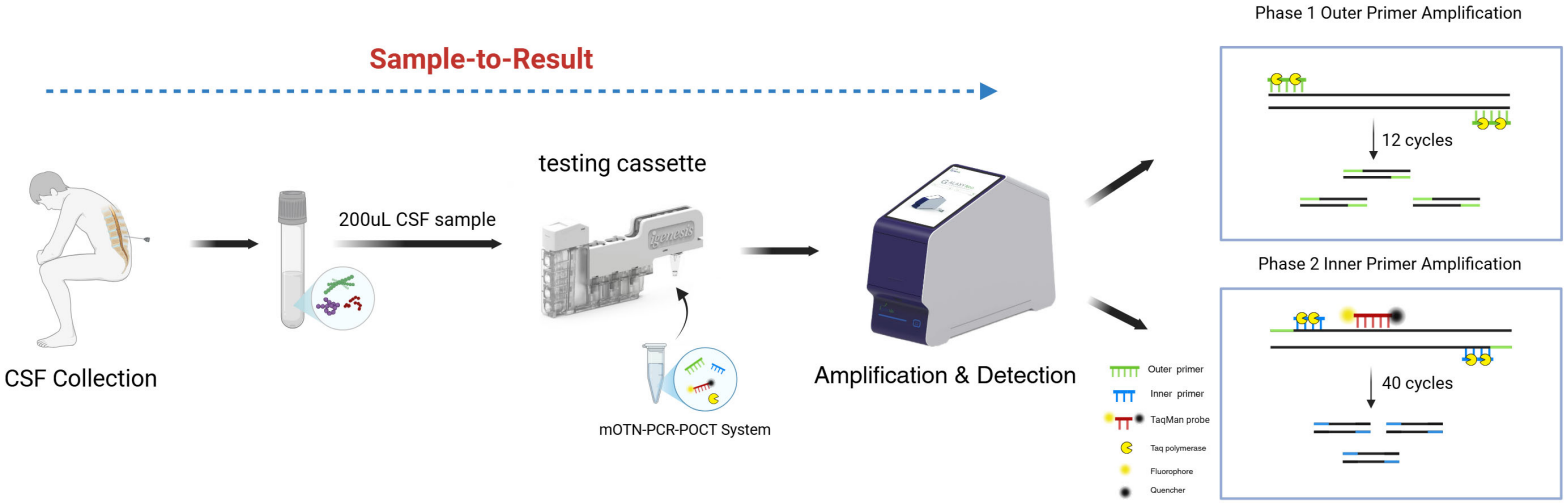
Flowchart of the mOTN-PCR-POCT method. CSF, cerebrospinal fluid; mOTN-PCR, multiplex one-tube nested real-time polymerase chain reaction; POCT, point-of-care testing. This figure was created by BioRender.

SPN, GBS, and GAS reference strains were provided by the Microbiology Department of Hebei General Hospital. Other reference strains used for specific validation were sourced from the strain repository maintained by our laboratory. This study retrospectively collected 109 clinical residual samples from Hebei General Hospital between February 26, 2025, and February 20, 2026 (sample types and distribution are detailed in **Table 1**). All positive samples were confirmed by mass spectrometry at Hebei General Hospital. Following collection, all samples were placed in sterile tubes, transported on ice to the Central Laboratory of the National Center for Viral Disease Control and Prevention, Chinese Center for Disease Control and Prevention, and stored at −80 °C. This study was conducted in accordance with national ethical regulations and approved by the Hebei General Hospital Ethics Committee. Manual nucleic acid extraction was performed on reference strains and selected clinical samples using the FastPure^®^ Microbiome DNA Isolation Kit (Vazyme, Nanjing, China) following the manufacturer’s instructions. The concentration of extracted DNA from reference strains was measured using a Qubit^®^ Double-Stranded DNA High Sensitivity Assay Kit (Thermo Fisher Scientific, MA, United States) on a Qubit 2.0 Fluorometer (Life Technologies, United States). Subsequently, the quantified DNA stock solution was serially diluted 10-fold with 1× TE buffer for sensitivity evaluation of the method as described in Section 2.4. All diluted DNA templates were aliquoted and stored at −80 °C until use. The POCT instrument (ENucFlow-PS2 Fully Automated Integrated Nucleic Acid Extraction and Detection System) was provided by WeEasyBio (Shanghai, China). This instrument performs fully automated nucleic acid extraction based on the magnetic bead method, combined with ultrasonic and thermal lysis, and automatically completes all steps from nucleic acid extraction to real-time PCR analysis within a closed system (**Figure 1**).

**Table 1:** Composition and distribution of the clinical samples included in this study (n=109).

Category	Specimen type	Culture result	Number (n)
CSF	CSF	SPN (Positive)	6
		Culture-negative	30
Non-CSF	Sputum	SPN (Positive)	7
		Culture-negative	20
	Vaginal swab	GBS (Positive)	4
		Culture-negative	20
	Throat swab	GAS (Positive)	2
		Culture-negative	20
Total			109

The culture-negative CSF samples served as negative controls and were used for background analysis in this study. SPN, *Streptococcus pneumoniae*; GBS, *Streptococcus agalactiae*; GAS, *Streptococcus pyogenes*.

### 2.2 Design of mOTN-PCR primers and probes and plasmid construction

SPN *LytA* gene, GBS *sip* gene, and GAS *CsrRS* gene data were downloaded from the National Center for Biotechnology Information (NCBI) database (Ganaie et al., 2015; Finn et al., 2021; Ji et al., 2024). Bioedit software was used for sequence alignment, and highly conserved regions were identified for primer and probe design. Nested PCR amplification required one pair of outer primers, one pair of inner primers, and one TaqMan probe. The outer primers were used for the first-round preamplification and designed using Primer 6 software; their specificity was evaluated via the NCBI BLAST tool. Oligo 7 software was employed to analyze and avoid primer dimers, hairpin structures, and mismatches. The inner primers and probe were used for the second round of real-time PCR detection. They were derived from published literature (Bergh et al., 2004; Ganaie et al., 2015; Oeser et al., 2020), with their target sites located within the amplification products of the outer primers. All primers and probes were synthesized and purified by BiOligo Biotechnology (Shanghai, China). The sequence information is shown in **Table 2**.

**Table 2:** Primer and probe sequences used in this study.

Species	Primer/probe	Sequence (5’-3’)	Source
SPN	SPN-outer-F	CTGGGACGTTGGGGGCGGTTGGAATGCTGAGACCTATG	This study
	SPN-outer-R	ACTGCTCACGGCTAATGCCCCATTTTGCCAAGTAAGGGT	This study
	SPN-inner-F	ACGCAATCTAGCAGATGAAGCA	(Ganaie et al., 2015)
	SPN-inner-R	TCGTGCGTTTTAATTCCAGCT	(Ganaie et al., 2015)
	SPN-Probe^a^	VIC-GCCGAAAACGCTTGATACAGGGAG-BHQ1	(Ganaie et al., 2015)
GBS	GBS-outer-F	ACGACGTGGACAGCACGTACTGTTTCAGAGGTAAAGGCT	This study
	GBS-outer-R	TGGCGAAACAATCGTTGTTGCTGCTTCTGGTGTCATACCTTCCGA	This study
	GBS-inner-F	ATCCTGAGACAACACTGACA	(Bergh et al., 2004)
	GBS-inner-R	TTGCTGGTGTTTCTATTTTCA	(Bergh et al., 2004)
	GBS-Probe^a^	ROX-ATCAGAAGAGTCATACTGCCACTTC-BHQ2	(Bergh et al., 2004)
GAS	GAS-outer-F	CCAGAGATGGATGGTTTTGAAGTGACCCGTCGTTTGCA	This study
	GAS-outer-R	TCTTGACGGCGGAAAATAGCACGAATACGGGCAAGTAGT	This study
	GAS-inner-F	TTATGGATGTGGTTGCAGGT	This study
	GAS-inner-R	CGGGCAAGTAGTTCTTCAATGG	(Oeser et al., 2020)
	GAS-Probe^a^	FAM-CGGTGCAGACGACTATATTGTTAAACC-BHQ1	(Oeser et al., 2020)

a Probe modifications: VIC, 6-phosphoramidite; ROX, 5-carboxy-X-rhodamine; FAM, 6-carboxyfluorescein; BHQ, black hole quencher; SPN, *Streptococcus pneumoniae*; GBS, *Streptococcus agalactiae*; GAS, *Streptococcus pyogenes*.

Fragments of the *LytA*, *sip*, and *CsrRS* genes from SPN, GBS, and GAS (324 bp, 420 bp, and 327 bp, respectively) were cloned into the pUC57 vector provided by TsingKe Biotech Corp. (Beijing, China). Plasmid concentrations were quantified using the Qubit^®^ Double-Stranded DNA High Sensitivity Assay Kit in conjunction with the Qubit 2.0 Fluorometer. Plasmid copy number was calculated using the following formula: Plasmid copy number (copies/μL) = [6.02 × 10^23^ × plasmid concentration (ng/μL) × 10^-9^]/[plasmid length × 660] (Nie et al., 2022). Finally, the plasmid was serially diluted 10-fold in 1× TE buffer to generate a panel of standards at concentrations ranging from 10^0^ to 10^5^ copies/μL. Aliquots were stored at −80 °C.

### 2.3 Establishment and optimization of the mOTN-PCR detection

To establish an optimized multiplex Nested PCR reaction system, multiple parameters, including real-time PCR enzymes, annealing temperatures, and primer/probe concentration ratios, underwent systematic optimization. To identify the most suitable enzyme for multiplex detection, two commercial premixes, Taq Pro HS DNA Polymerase and Taq Pro U+ Multiple Probe qPCR Mix (both from Vazyme, Nanjing, China), were compared. Preliminary results demonstrated that Taq Pro U+ Multiple Probe qPCR Mix exhibited superior performance, yielding lower Ct values for low-copy templates and greater tolerance to multiplex reaction conditions. Therefore, this enzyme was selected for all subsequent mOTN-PCR assays.

To determine the optimal annealing temperature, this study conducted gradient PCR experiments at temperatures ranging from 51 °C to 72 °C. The gradient PCR was performed in a 20 μL reaction volume containing 10 μL of 2× Taq Pro U+ Multiple Probe qPCR Mix, 0.4 μL of 10 μM outer primers (or inner primers, depending on the experimental group), 1 μL of recombinant plasmid DNA (10^4^ copies/μL) as template, and nuclease-free water to a final volume of 20 μL, resulting in a final primer concentration of 200 nM. The thermal cycling conditions were as follows: 95 °C for 3 minutes, followed by 40 cycles of 95 °C for 15 s and a gradient annealing/extension step from 51 °C to 72 °C for 45 s. Two parallel experiments were performed for each temperature and each target: one containing only the outer primer and template, and another containing only the inner primer and template. This strategy aimed to optimize the annealing temperature for the outer primers, ensuring the inner primers did not bind to the DNA template during the outer primer reaction phase while maintaining high amplification efficiency for the inner primers in subsequent reactions. The final amplified products were analyzed via 3% agarose gel electrophoresis and stained with GelRed (Biotium, USA) for visualization under UV illumination.

Based on our group’s prior research, the highest amplification efficiency for the inner primer reaction was achieved when the inner-to-outer primer concentration ratio exceeded 10:1, and the annealing temperature difference between inner and outer primers was maintained at 10 °C or higher (Shen et al., 2018; Zhao et al., 2019; Zhang et al., 2020). Building upon this foundation, the ratio of primers to probes for different targets was adjusted to ensure efficient amplification for each target. The final composition of the mOTN-PCR reaction system (20 μL) includes: 10 μL of 2× Taq Pro U+ Multiple Probe qPCR Mix; final concentrations of 5 nM for SPN and GAS outer primers, 15 nM for GBS outer primers, and 200 nM for all inner primers and probes; 1 μL of DNA template (with concentrations varying depending on the specific assays, as detailed below); and nuclease-free water was added to a final volume of 20 μL. The probes were labeled with distinct fluorescent reporter groups: SPN (*LytA*)-VIC, GBS (*sip*)-ROX, and GAS (*CsrRS*)-FAM, enabling triple detection in a single tube. The amplification program is as follows: 95 °C pre-denaturation for 5 minutes; 12 cycles of 95 °C for 15 s, 68 °C for 45 s (external primers are nearly depleted in the first stage), followed by 40 cycles at 95 °C for 15 s, 55 °C for 45 s. Throughout the detection process, nuclease-free water served as the negative control, while recombinant plasmids and reference bacterial strain nucleic acids functioned as positive controls. All real-time PCR assays in this study were designed and reported in accordance with the MIQE guidelines to ensure reproducibility and transparency (Bustin et al., 2009).

### 2.4 Sensitivity, repeatability, and specificity evaluation

To evaluate the sensitivity and reproducibility of the mOTN-PCR assay, recombinant plasmids of SPN, GBS, and GAS (gradient diluted from 10^0^ to 10^5^ copies/μL) and nucleic acids from reference strains (gradient diluted from 10^-7^ to 10^-1^ ng/μL) were used as templates for detection. Each dilution level was tested in eight replicates, with nuclease-free water serving as the negative control. For specificity validation, 18 microorganisms associated with CNS infections were included. Beyond the target organisms (SPN, GBS, and GAS), 15 additional common pathogens (detailed in **Supplementary Table 1**) were selected to assess cross-reactivity of the mOTN-PCR assay. To further clarify the method’s sensitivity advantage, parallel testing of mOTN-PCR and conventional real-time PCR was performed using the same templates from the same source and at the same concentrations. The primers and probes used for conventional triplex real-time PCR were the same as the inner primers and probes used in the mOTN-PCR assay (detailed in **Table 2**). The real-time PCR reaction system (20 μL) contained: 10 μL of 2× Taq Pro U+ Multiple Probe qPCR Mix, 0.4 μL each of the forward primer, reverse primer, and probe for each target (resulting in a final concentration of 200 nM for each), 1 μL of DNA template, and nuclease-free water was added to a final volume of 20 μL. A conventional real-time PCR reaction cycle threshold (Ct) below 38 was considered positive. The amplification program for the triple real-time PCR was as follows: 95 °C for 3 minutes; 40 cycles at 95 °C for 15 s, 55 °C for 45 s. By comparing the detection results of the two methods, the improvement in sensitivity of mOTN-PCR could be directly assessed.

### 2.5 Preparation of quantitative simulated CSF samples

After thawing SPN, GBS, and GAS reference strains stored at −80 °C, they were streaked onto Columbia blood agar plates (Hopebiol, Qingdao, China) and incubated at 37 °C in a 5% CO_2_ incubator (Thermo Fisher Scientific, USA) for 24 hours. After incubation, single colonies were picked and inoculated into Brain Heart Infusion (BHI) broth: SPN was incubated statically at 37 °C, 5% CO_2_ for 8 hours; GBS and GAS were incubated overnight at 37 °C, 220 rpm with shaking. After incubation, 1 mL of bacterial suspension was centrifuged, supernatant discarded, and the pellet washed twice with 1 mL PBS (GIBCO, USA). The pellet was resuspended in 1 mL PBS and vortex-mixed to prepare a bacterial serial dilution. Colony counts were then performed using the drop plate method. Brief procedure: Perform a 10-fold serial dilution of the bacterial suspension. Take 10 μL from each dilution and dispense onto pre-marked areas on Columbia blood agar plates, with 3 replicates per dilution. After drying, incubate plates upside down at 37 °C for 18–24 hours until visible colonies develop in the original droplet areas (small circular zones). Select dilutions yielding countable colonies (3–30 colonies) and calculate the original bacterial concentration using the following formula: Colony-forming units (CFU/mL) = Number of colonies/Dilution factor × Plating volume (10 μL)(Naghili et al., 2013). The principle of the drop plate method is shown in **Supplementary Figure 1**. Next, add the quantified bacterial suspension to 1 mL of healthy human CSF to prepare simulated CSF samples with final concentrations of 5, 10, 20, 50, 100, 200, 500, and 1000 CFU/mL. Simultaneously, prepare PBS bacterial suspension samples at the same concentrations and incubate them overnight on blood agar plates at 37 °C to accurately calculate the actual CFU concentrations in the simulated CSF samples. The limit of detection (LOD) for simulated CSF samples was defined as the lowest concentration at which all three replicates tested positive in at least three independent experiments.

### 2.6 Nucleic acid extraction from simulated samples and clinical samples

Manual extraction and fully automated nucleic acid extraction methods were employed to extract nucleic acids from simulated and clinical samples, serving as templates for mOTN-PCR and real-time PCR. For manual extraction, procedures were strictly followed according to the nucleic acid extraction kit instructions. Brief procedure: 400 µL of simulated sample or clinical sample underwent sequential steps, including lysis, binding, and washing for nucleic acid purification. After elution with 50 µL of elution buffer, the purified nucleic acid was collected in a 1.5 mL sterile centrifuge tube and stored at −80 °C. For fully automated nucleic acid extraction, the POCT instrument and its dedicated testing cassettes are employed, with each testing cassette functioning as a standalone “PCR laboratory”. Nucleic acid extraction is automatically performed based on the classic magnetic bead method. Briefly, 200 µL of the sample was aspirated into the testing cassette containing a pre-loaded 15 µL mOTN-PCR reaction system, and the entire process from lysis to data analysis will be automatically completed.

### 2.7 Detection of simulated samples and clinical samples

Simulated samples of SPN, GBS, and GAS were submitted to two extraction protocols: the manual kit (FastPure^®^ Microbiome DNA Isolation Kit) and the POCT instrument. After that, both mOTN-PCR and real-time PCR were performed on samples derived from the two extraction methods, and the sensitivity and specificity results using different extraction protocols were compared for target pathogens detection in CSF. For the 36 clinical CSF samples and 73 non-CSF clinical samples, two parallel testing workflows were performed: (i) manual nucleic acid extraction using the FastPure^®^ Microbiome DNA Isolation Kit followed by conventional triplex real-time PCR (using the 2× Taq Pro U+ Multiple Probe qPCR Mix as detailed in Section 2.4); and (ii) fully automated nucleic acid extraction using the POCT instrument followed by mOTN-PCR. The goal was to compare the clinical performance and operational workflow of the mOTN-PCR-POCT system against the conventional laboratory method (manual extraction + real-time PCR).

### 2.8 Statistical analysis

All real-time PCR amplification curves were visualized and analyzed using GraphPad Prism version 10.1.2 (GraphPad Software, USA). Statistical analysis was performed using SPSS 26.0 software (IBM, USA). The Kappa test was used to assess the consistency between the mOTN-PCR method and the real-time PCR method. In addition, McNemar’s exact test was used to compare the paired detection rates of the mOTN-PCR-POCT and conventional real-time PCR assays for each pathogen separately. Due to the limited number of positive samples, Fisher’s combined test was applied to the individual *P*-values to obtain an overall assessment across the three pathogens. A *P*-value < 0.05 was considered statistically significant. Diagnostic performance, including sensitivity, specificity, positive predictive value (PPV), and negative predictive value (NPV), was calculated for both methods using culture results as the reference standard. The 95% confidence intervals (CIs) for these metrics were computed using the exact binomial method.

## 3 Results

### 3.1 Optimization of the mOTN-PCR detection method

Through optimization of key reaction parameters, optimal conditions for mOTN-PCR detection of SPN, GBS, and GAS were ultimately established. Results indicated that the optimal concentrations for inner primers and probes were identical for all three targets. During outer primer concentration optimization, SPN and GAS required the same optimal outer primer concentration, while GBS required a concentration three times that of the other two. Thus, the final outer primer ratio was established as SPN: GAS: GBS = 1:1:3.

The agarose gel electrophoresis results (**Supplementary Figure 2**), using 10^4^ copies/μL recombinant plasmids as templates, demonstrate that the outer primers achieve effective amplification under different annealing temperatures. SPN exhibited specific amplification of the outer primer without non-specific inner primer products at annealing temperatures of 68.2-72 °C. GBS exhibited a similar pattern at 70.1–72 °C, although a faint but specific inner primer band was visible at 68.2 °C, subsequent rigorous validation experiments confirmed that this faint inner band at 68.2 °C had no negative impact on the overall performance of the assay. Similar to SPN, GAS demonstrated specific amplification without non-specific products at 66.3-72 °C. Separately, the annealing temperature of the inner primers was also optimized via gradient PCR. As shown in **Supplementary Figure 2**, robust and highly specific amplification with clear target bands was achieved for all three targets at annealing temperatures of 51 to 64.4 °C. To maximize amplification efficiency and clinical detection sensitivity for low-load CSF samples, we strategically selected the annealing temperatures that prioritize the balance between amplification efficiency (sensitivity) and specificity. Furthermore, as outlined in our assay design principles (Section 2.3), maintaining an annealing temperature difference of 10 °C or higher between inner and outer primers is critical for achieving optimal nested reaction efficiency. Based on the optimal experimental observations and these established theoretical requirements, the final triple mOTN-PCR reaction program was determined as follows: outer primer annealing temperature 68 °C, inner primer annealing temperature 55 °C (these two temperatures represent the closest practical integer settings on the instrument, selected based on the optimal values of 68.2 °C and 54.8 °C). Additionally, the first-round preamplification was extended from 10 to 12 cycles to ensure mOTN-PCR amplification efficiency.

Finally, for real-time PCR enzyme selection, this study employed the Taq Pro U+ Multiple Probe qPCR Mix, which is optimized for multiplex PCR systems. This enzyme significantly enhances amplification performance, specificity, and detection sensitivity for low-copy genes under multiplex conditions, while demonstrating excellent tolerance to contaminants and versatility across multiple detection scenarios.

### 3.2 Sensitivity, specificity, and repeatability of the mOTN-PCR detection method

The sensitivity of the mOTN-PCR method for detecting SPN, GBS, and GAS was evaluated using recombinant plasmids and diluted reference bacterial strains. In eight replicate experiments (**Supplementary Table 2**, **Supplementary Figure 3**), the detection limits of mOTN-PCR for SPN, GBS, and GAS recombinant plasmids were 5, 10, and 10 copies/μL, respectively, while the detection limits for nucleic acids from the corresponding reference strains were 10^-5^, 10^-6^, and 10^-6^ ng/μL, respectively. In contrast, conventional real-time PCR exhibited LODs of 10^2^ copies/μL for all three recombinant plasmids, and 10^-4^, 10^-5^, and 10^-5^ ng/μL for the reference bacterial strain nucleic acid (**Supplementary Table 3**, **Supplementary Figure 4**). In all experiments, no amplification curve was observed for the negative control, and repeat testing yielded consistent results.

In specificity testing, the method produced distinct amplification curves only for the target pathogens SPN, GBS, and GAS, as presented in the **Supplementary Table 1**. No observable amplification signals were detected for the remaining 15 non-target pathogens (including *Streptococcus mitis*, *Streptococcus oralis*, *Streptococcus anginosus*, etc.), demonstrating excellent specificity and no cross-reactivity with other pathogens.

### 3.3 Evaluation and comparison of mOTN-PCR and real-time PCR in simulated samples

**Figure 2:**
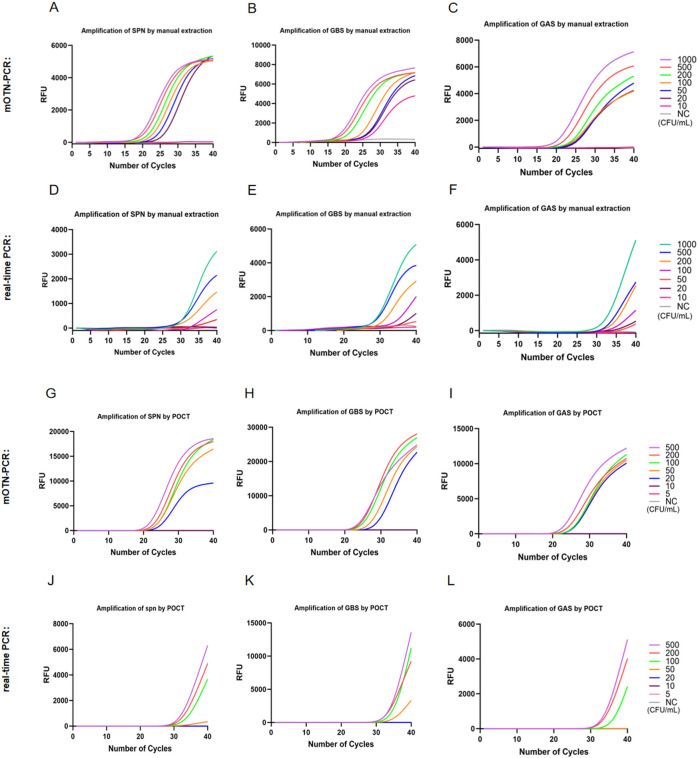
Comparison of sensitivity between mOTN-PCR and real-time PCR for the detection of SPN, GBS, and GAS in simulated CSF samples. The LOD of mOTN-PCR and real-time PCR for three pathogens was evaluated using nucleic acids extracted manually (sample concentration: 10–1000 CFU/mL) and via fully automated extraction with a POCT instrument (sample concentration: 5–500 CFU/mL). **(A–C)** mOTN-PCR results after manual extraction; **(D–F)** real-time PCR results after manual extraction; **(G–I)** mOTN-PCR results after POCT fully automated extraction; **(J–L)** real-time PCR results after POCT fully automated extraction. RFU, relative fluorescence units; NC, negative control; SPN, *Streptococcus pneumoniae*; GBS, *Streptococcus agalactiae*; GAS, *Streptococcus pyogenes*; mOTN-PCR, multiplex one-tube nested real-time polymerase chain reaction; real-time PCR, real-time polymerase chain reaction; POCT, point-of-care testing.

For nucleic acids extracted manually from simulated samples, the LOD for mOTN-PCR in detecting SPN, GBS, and GAS simulated CSF (10–1000 CFU/mL) were 20, 10, and 20 CFU/mL, respectively (**Figures 2A–C**), whereas conventional real-time PCR exhibited detection limits of 200, 100, and 100 CFU/mL, respectively (**Figures 2D–F**). On the POCT instrument, mOTN-PCR achieved a LOD of 20 CFU/mL for simulated CSF (5–500 CFU/mL) containing SPN, GBS, and GAS (**Figures 2G–I**), whereas conventional real-time PCR had LODs of 100, 50, and 100 CFU/mL, respectively (**Figures 2J–L**). These results consistently demonstrate that mOTN-PCR exhibits significantly higher detection sensitivity than conventional real-time PCR under both manual nucleic acid extraction and POCT fully automated extraction conditions. The reproducibility of mOTN-PCR and real-time PCR for simulated CSF samples was assessed, and the detailed detection rates are presented in **Supplementary Table 4** (manual extraction, 10 to 1000 CFU/mL) and **Supplementary Table 5** (POCT fully automated extraction, 5 to 500 CFU/mL).

### 3.4 Evaluation and comparison of mOTN-PCR and real-time PCR in clinical samples

A total of 109 clinical samples (19 culture-positive and 90 culture-negative) were analyzed, including 36 CSF samples and 73 non-CSF samples. Each sample was tested using two methods: conventional real-time PCR following manual nucleic acid extraction and the mOTN-PCR-POCT assay (**Table 3**). Results showed that among the 19 culture-positive samples, 16 (14.7%) tested positive by both real-time PCR and mOTN-PCR-POCT. Additionally, 3 (2.8%) culture-positive samples were undetected by real-time PCR but were positive by mOTN-PCR-POCT. The remaining 82.6% of culture-negative samples were negative in both methods. The mOTN-PCR-POCT results of 109 samples were compared with the real-time PCR results, and the Kappa values were 0.906, 0.853, and 1.000, respectively, with a *P*-value < 0.05. This indicates strong concordance between the two methods, validating the reliability of the mOTN-PCR-POCT method for clinical practice.

**Table 3:** Detection of SPN, GBS, and GAS in clinical samples.

Species	mOTN-PCR		Real-time PCR		Kappa
	Positive	Negative	Positive	Negative	
SPN	13	96	11	98	0.906
GBS	4	105	3	106	0.853
GAS	2	107	2	107	1.000

SPN, *Streptococcus pneumoniae*; GBS, *Streptococcus agalactiae*; GAS, *Streptococcus pyogenes*; mOTN-PCR, multiplex one-tube nested real-time polymerase chain reaction; real-time PCR, real-time polymerase chain reaction.

To further quantify the superiority of the mOTN-PCR-POCT, we performed McNemar’s exact test for each pathogen separately. The results showed that mOTN-PCR-POCT detected two additional SPN-positive cases (*P* = 0.50) and one additional GBS-positive case (*P* = 1.00) compared to real-time PCR, while the results for GAS were identical (*P* = 1.00). Although these individual *P*-values did not reach statistical significance due to the limited number of positive samples, Fisher’s combined test across the three pathogens also yielded a non-significant overall *P*-value of 0.986. Nevertheless, the detection of three additional culture-positive cases (representing 15.8% of all positives) highlights the enhanced sensitivity of the mOTN-PCR-POCT assay in a clinically relevant context. Using culture results as the reference standard, mOTN-PCR-POCT demonstrated superior diagnostic performance, achieving 100% sensitivity (19/19, 95% CI: 82.4–100%) and 100% specificity (90/90, 95% CI: 96.0–100%). In contrast, conventional real-time PCR showed a sensitivity of 84.2% (16/19, 95% CI: 60.4–96.6%) and 100% specificity. The detailed diagnostic performance metrics for both methods are presented in **Supplementary Table 6**.

## 4 Discussion

Although POCT instruments have significantly advanced rapid molecular diagnostics, their application to CSF pathogen detection remains challenging. Taking the widely adopted GeneXpert system (Cepheid, USA) as an example, although its fully automated, closed-tube design is consistent with the “sample-in, result-out” testing concept, this system’s sensitivity in CSF testing remains limited, particularly for specimens with extremely low pathogen loads or those collected after antimicrobial therapy (Ninove et al., 2011; Wang et al., 2024; Liu et al., 2024; Heemskerk et al., 2018). Meanwhile, multiplex PCR-based systems like FilmArray and QIAstat-Dx enable multi-target screening but rely on fixed detection panels, lacking the flexibility to adapt to local epidemiological needs (Le Bars et al., 2023). Additionally, some recent automated systems (such as NeuMoDx288) still require the manual transfer of nucleic acid extracts during the workflow, failing to achieve true end-to-end integration (Luzius et al., 2025). Finally, these POCT systems typically rely on proprietary large-scale instruments and reagents, which are costly and limit their application in primary care settings, emergency departments, or at the bedside (Matthes et al., 2023).

To overcome these limitations, we developed and optimized the mOTN-PCR-POCT assay. This system integrates the high sensitivity of Nested PCR with a fully automated, enclosed POCT platform. Its core innovation lies in the high integration of the entire testing workflow within a sealed testing cassette, sequentially and automatically executing the following key steps: first, samples undergo ultrasonic and thermal lysis to fully release nucleic acids; then, nucleic acid extraction and purification are performed within the testing cassette based on the classic magnetic bead method; subsequently, purified nucleic acids are transferred to the amplification reaction zone via physical displacement; finally, the instrument controls variable-temperature amplification while simultaneously collecting fluorescence signals, automatically generating amplification curves and analytical results. By eliminating the cumbersome manual “step-by-step” procedures of traditional molecular workflows (such as the FastPure^®^ Microbiome DNA Extraction protocol) (Jin et al., 2024; Sharma and Pokharel, 2025; Wang et al., 2025), this “sample-in, result-out” system fundamentally eliminates aerosol contamination and cross-contamination between samples. It integrates extraction and amplification into a fully automated process lasting approximately 2 hours, significantly reducing the 2–3 day diagnostic delays associated with traditional microbial culture. Overall, this method achieves “one-click” testing by integrating three aspects: time (process reduction), space (liberation from fixed laboratories), and manpower (simplified operation). It aligns closely with practical clinical demand for rapid, convenient, and reliable diagnostics.

Analytically, the assay demonstrated high reliability, achieving a LOD as low as 20 CFU/mL for SPN, GBS, and GAS in simulated CSF. This high level of analytical sensitivity successfully translates into significant clinical utility, enabling the detection of culture-positive cases that conventional real-time PCR fails to identify. A key feature of the mOTN-PCR-POCT system is its semi-open cassette architecture and modular primer-probe combination strategy. Highly specific nested primers and TaqMan probes were designed targeting conserved regions of CSF pathogens, which can be manually added to the reaction wells of the testing cassette before sample loading, thereby allowing for flexible adaptation to local epidemiological characteristics or emerging pathogens. Operationally, the workflow requires no complex pretreatment: simply add 200 μL of CSF sample to the pre-loaded reagent testing cassettes to initiate fully automated testing, which can be easily performed by ordinary medical personnel. This minimal sample volume requirement is particularly advantageous for pediatric patients or situations where lumbar puncture is difficult.

Beyond performance, the cost-effectiveness and accessibility of the mOTN-PCR-POCT system facilitate its widespread clinical adoption. The testing cassettes employed in this study are amenable to standardized and scalable manufacturing, eliminating reliance on large, expensive instruments. The equipment cost of our platform is approximately $42,000, significantly lower than that of the BioFire FilmArray system ($100,000–150,000) and the Cepheid GeneXpert system ($50,000–80,000). Regarding the per-test cost, following large-scale production, the cost of our method can be reduced to $4–7 per test, offering a significant advantage over FilmArray ($100–200) and GeneXpert ($30–50). This exceptional cost-efficiency, combined with the instrument’s compact physical dimensions (448 mm × 178 mm × 331 mm), lightweight design (15.6 kg), and the ability to store testing cartridges at room temperature, makes this system an ideal diagnostic tool for primary care hospitals and resource-limited areas.

Given the recognized high sensitivity of nested PCR (Bestetti et al., 2001; Wang et al., 2020), we employed a highly systematic, multi-tiered evaluation framework—from gradient-diluted plasmids to authentic clinical specimens—ensuring a realistic assessment of the assay’s clinical applicability. Nevertheless, this study has certain limitations. First, absolute quantification is currently precluded because the two-round amplification mechanism inherently disrupts the linear relationship between initial template concentration and second-round Ct values. Second, the clinical sample size included in this study was limited, and the pathogen spectrum primarily focused on SPN, GBS, and GAS. This is insufficient for comprehensively evaluating the method’s diagnostic performance in broader populations, diverse pathogen prevalence patterns, and complex clinical settings. Future multi-center prospective studies with larger cohorts of positive samples are warranted to further validate the assay’s diagnostic accuracy across diverse populations and settings. This would establish it as a reliable point-of-care tool for early diagnosis of bacterial meningitis in emergency departments, bedside settings, and primary healthcare facilities.

## Data Availability

The datasets presented in this study can be found in online repositories. The names of the repository/repositories and accession number(s) can be found in the article/**Supplementary Material**.
