# Supplementary material for: A nested PCR-based point-of-care multiplex test for detection of bacterial pathogens in cerebrospinal fluids
Source: Front Cell Infect Microbiol. 2026 Apr 14;16:1781397. doi: 10.3389/fcimb.2026.1781397 (PMC13121276; doi:10.3389/fcimb.2026.1781397)
Supplement: Supplementary file 1 [file Supplementaryfile1.docx]

Supplementary material

# Supplementary Figures and Tables

## Supplementary Figures

| **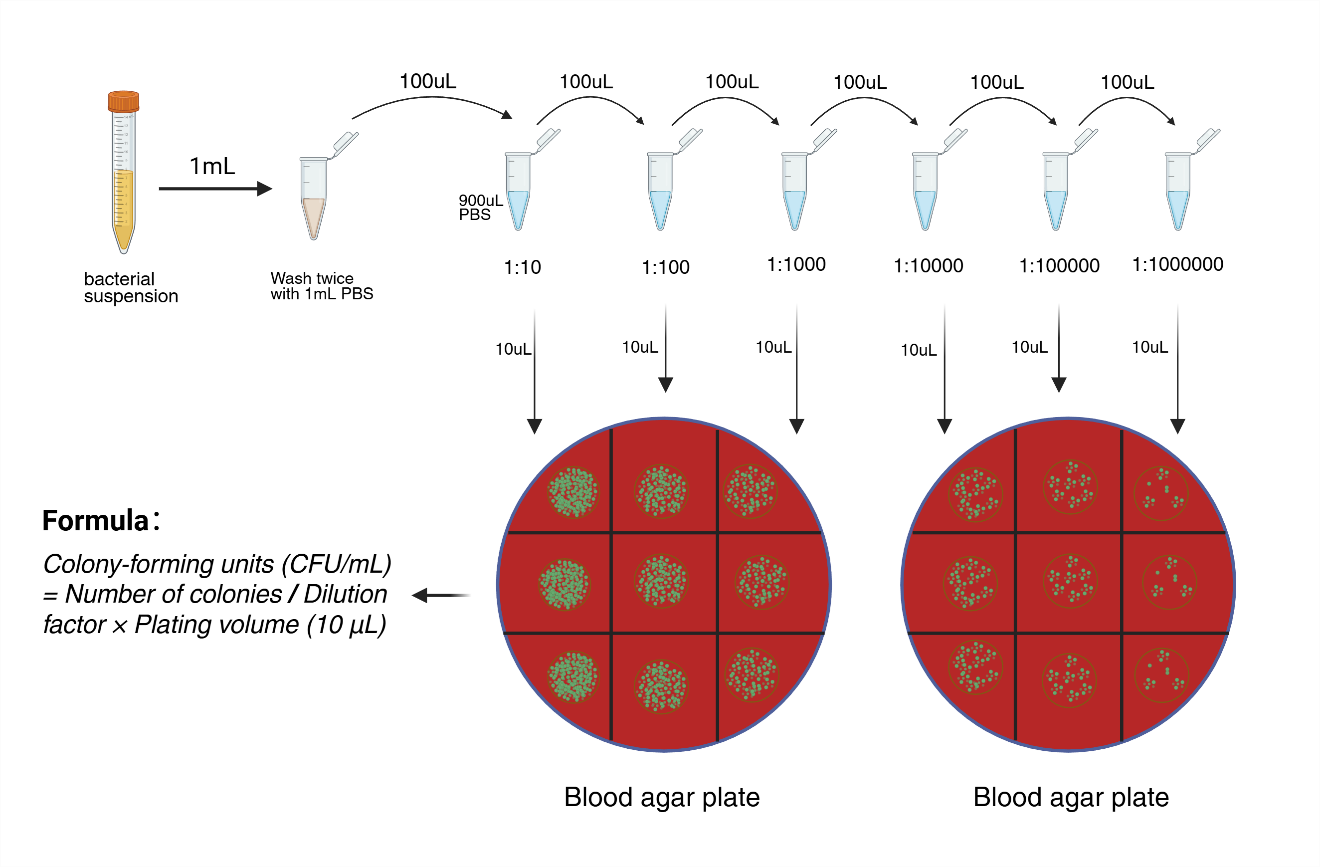** |
| --- |
| **Supplementary Figure 1.** The principle of the drop plate method. Three parallel samples were prepared for each dilution level, and the average colony count was used for calculation. This figure was created by BioRender. |

| 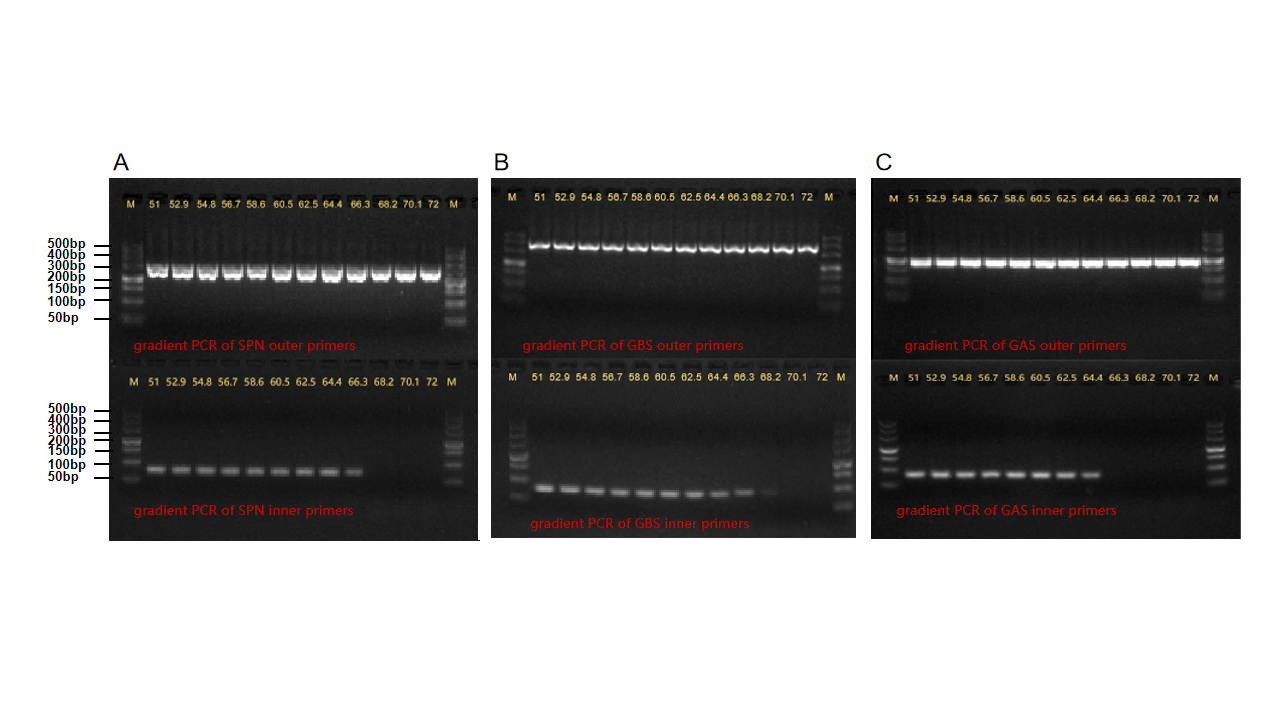 |
| --- |
| **Supplementary Figure 2.** Determination of optimal annealing temperatures for all primers by gradient PCR using standard recombinant plasmids (10^4^ copies/μL). SPN, *Streptococcus pneumoniae*; GBS, *Streptococcus agalactiae*; GAS, *Streptococcus pyogenes*. M = 500 bp DNA markers. |

| 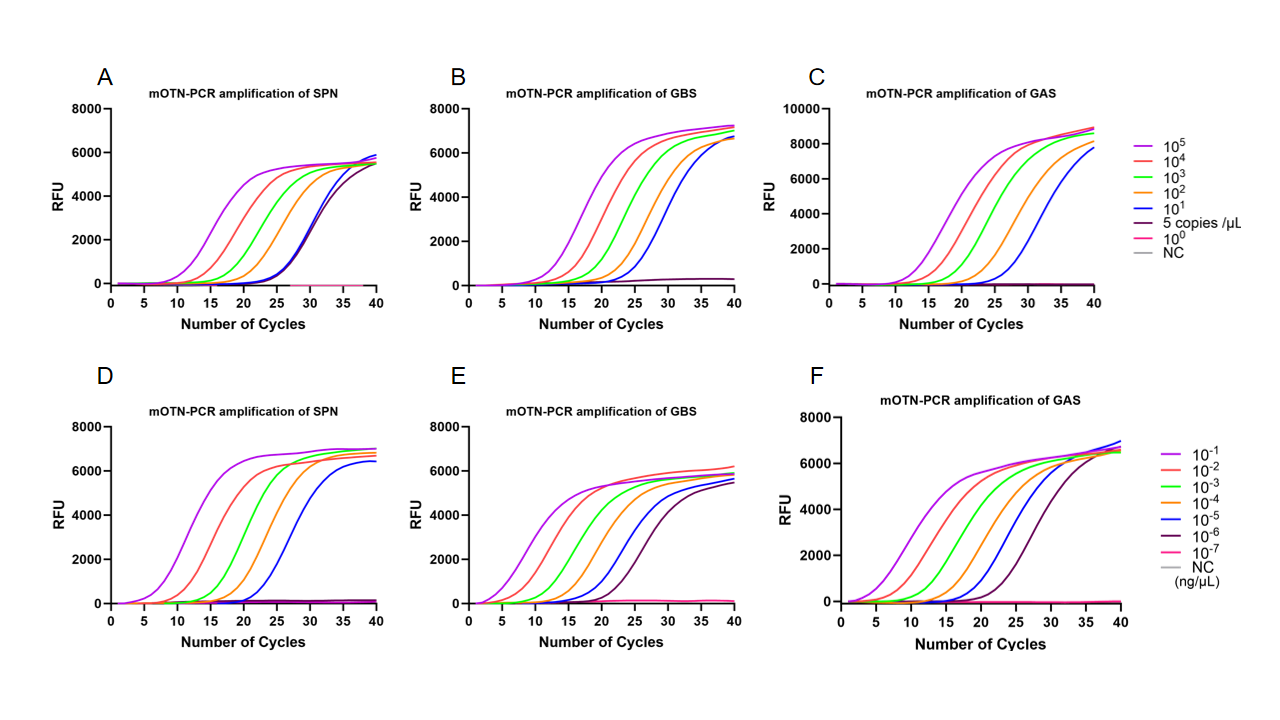 |
| --- |
| **Supplementary Figure 3.** Analytical sensitivity of the mOTN-PCR assay. The limit of detection (LOD) was assessed using recombinant plasmids (A-C) and nucleic acids of reference strains (D-F) for SPN, GBS, and GAS. The amplification curves shown are representative of eight independent replicates. RFU, relative fluorescence units; NC, negative control; SPN, *Streptococcus pneumoniae*; GBS, *Streptococcus agalactiae*; GAS, *Streptococcus pyogenes*; mOTN-PCR, multiplex one-tube nested real-time polymerase chain reaction. |

| 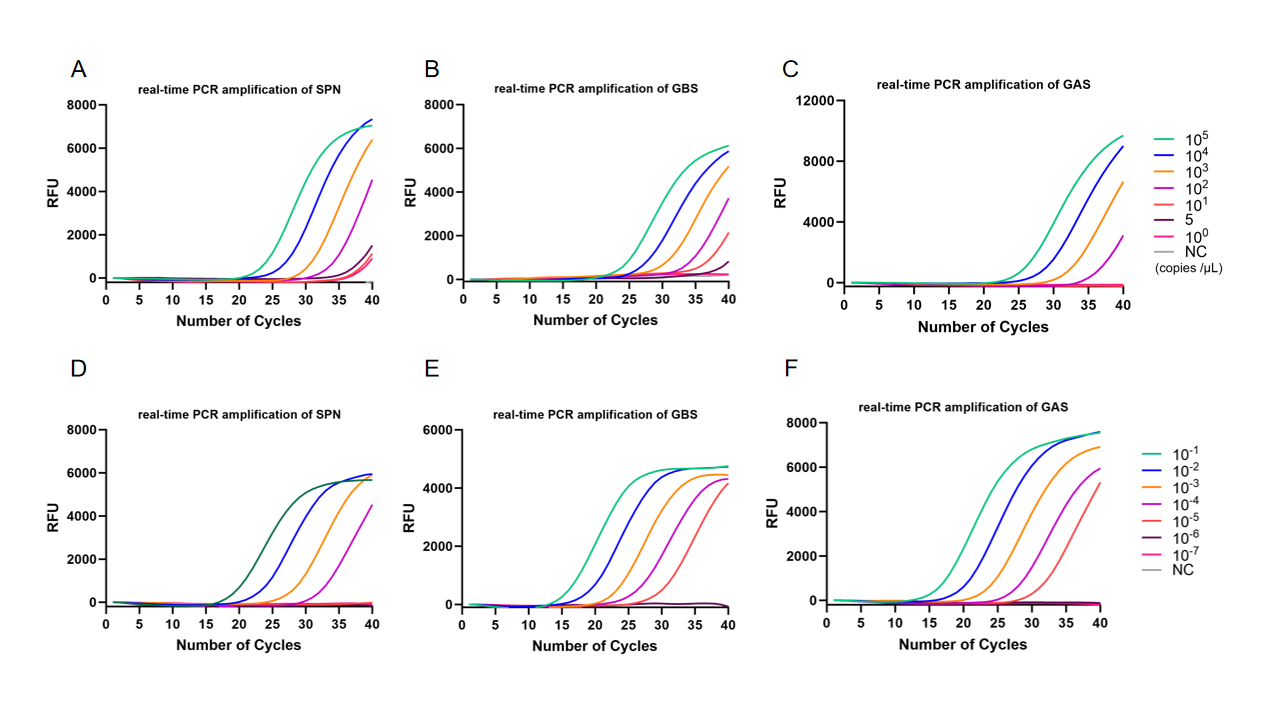 |
| --- |
| **Supplementary Figure 4.** Comparative analysis of the detection sensitivity for real-time PCR. The assay was evaluated with the same templates used for the mOTN-PCR assay. (A-C) Limits of detection (LOD) for SPN, GBS, and GAS recombinant plasmids. (D-F) LOD for nucleic acids from the corresponding reference bacterial strains. The amplification curves shown are representative of eight independent replicates. RFU, relative fluorescence units; NC, negative control; SPN, *Streptococcus pneumoniae*; GBS, *Streptococcus agalactiae*; GAS, *Streptococcus pyogenes*; real-time PCR, real-time polymerase chain reaction; mOTN-PCR, multiplex one-tube nested real-time polymerase chain reaction. |

## Supplementary Table

**Supplementary Table 1.** Microbial strains used in the specificity assay

| **Strains** | **Origin** | **SPN** | **GBS** | **GAS** |
| --- | --- | --- | --- | --- |
| *Streptococcus pneumoniae* | ATCC 49619 | Positive | Negative | Negative |
| *Streptococcus agalactiae* | ATCC 13813 | Negative | Positive | Negative |
| *Streptococcus pyogenes* | ATCC 19615 | Negative | Negative | Positive |
| *Streptococcus mitis* | Isolated strains | Negative | Negative | Negative |
| *Streptococcus oralis* | Isolated strains | Negative | Negative | Negative |
| *Streptococcus anginosus* | Isolated strains | Negative | Negative | Negative |
| *Haemophilus influenzae* | ATCC 49247 | Negative | Negative | Negative |
| *Klebsiella pneumoniae* | ATCC 11296 | Negative | Negative | Negative |
| *Escherichia coli* | ATCC 25922 | Negative | Negative | Negative |
| *Listeria monocytogenes* | ATCC 19111 | Negative | Negative | Negative |
| *Staphylococcus aureus* | ATCC 29213 | Negative | Negative | Negative |
| *Pseudomonas aeruginosa* | ATCC 27853 | Negative | Negative | Negative |
| *Neisseria meningitidis* | Isolated strains | Negative | Negative | Negative |
| *Enterococcus faecium* | Isolated strains | Negative | Negative | Negative |
| *Enterococcus faecalis* | Isolated strains | Negative | Negative | Negative |
| *Enterobacter cloacae* | Isolated strains | Negative | Negative | Negative |
| *Proteus mirabilis* | Isolated strains | Negative | Negative | Negative |
| *Stenotrophomonas maltophilia* | Isolated strains | Negative | Negative | Negative |

**Note:** SPN, *Streptococcus pneumoniae*; GBS, *Streptococcus agalactiae*; GAS, *Streptococcus pyogenes*.

**Supplementary Table 2.** The reproducibility of mOTN-PCR

| **Standard DNA (copies/μL)** | **SPN** | **GBS** | **GAS** | **Standard DNA (ng/μL)** | **SPN** | **GBS** | **GAS** |
| --- | --- | --- | --- | --- | --- | --- | --- |
| 10^5^ | 8/8^a^ | 8/8 | 8/8 | 10^-1^ | 8/8 | 8/8 | 8/8 |
| 10^4^ | 8/8 | 8/8 | 8/8 | 10^-2^ | 8/8 | 8/8 | 8/8 |
| 10^3^ | 8/8 | 8/8 | 8/8 | 10^-3^ | 8/8 | 8/8 | 8/8 |
| 10^2^ | 8/8 | 8/8 | 8/8 | 10^-4^ | 8/8 | 8/8 | 8/8 |
| 10^1^ | 8/8 | 8/8 | 8/8 | 10^-5^ | 8/8 | 8/8 | 8/8 |
| 5 | 8/8 | 7/8 | 7/8 | 10^-6^ | 6/8 | 8/8 | 8/8 |
| 10^0^ | 6/8 | 5/8 | 0/8 | 10^-7^ | 0/8 | 6/8 | 5/8 |

**Note:** ^a^The first number indicates the number of times a positive result occurred. The second number means that the experiment was repeated eight times. SPN, *Streptococcus pneumoniae*; GBS, *Streptococcus agalactiae*; GAS, *Streptococcus pyogenes*; mOTN-PCR, multiplex one-tube nested real-time polymerase chain reaction.

**Supplementary Table 3.** The reproducibility of real-time PCR

| **Standard DNA (copies/μL)** | **SPN** | **GBS** | **GAS** | **Standard DNA (ng/μL)** | **SPN** | **GBS** | **GAS** |
| --- | --- | --- | --- | --- | --- | --- | --- |
| 10^5^ | 8/8^a^ | 8/8 | 8/8 | 10^-1^ | 8/8 | 8/8 | 8/8 |
| 10^4^ | 8/8 | 8/8 | 8/8 | 10^-2^ | 8/8 | 8/8 | 8/8 |
| 10^3^ | 8/8 | 8/8 | 8/8 | 10^-3^ | 8/8 | 8/8 | 8/8 |
| 10^2^ | 8/8 | 8/8 | 8/8 | 10^-4^ | 8/8 | 8/8 | 8/8 |
| 10^1^ | 1/8 | 4/8 | 0/8 | 10^-5^ | 4/8 | 8/8 | 8/8 |
| 5 | 0/8 | 0/8 | 0/8 | 10^-6^ | 0/8 | 4/8 | 3/8 |
| 10^0^ | 0/8 | 0/8 | 0/8 | 10^-7^ | 0/8 | 0/8 | 0/8 |

**Note:** ^a^The first number indicates the number of times a positive result occurred. The second number means that the experiment was repeated eight times. SPN, *Streptococcus pneumoniae*; GBS, *Streptococcus agalactiae*; GAS, *Streptococcus pyogenes*; real-time PCR, real-time polymerase chain reaction.

**Supplementary Table 4.**Reproducibility of mOTN-PCR and real-time PCR for detecting SPN, GBS, and GAS in simulated CSF samples after manual nucleic acid extraction

| **Pathogen** | **Concentration (CFU/mL)** | **mOTN-PCR (Positive/Total)** | **real-time PCR (Positive/Total)** |
| --- | --- | --- | --- |
| SPN | 1000 | 3/3 | 3/3 |
|  | 500 | 3/3 | 3/3 |
|  | 200 | 3/3 | 3/3 |
|  | 100 | 3/3 | 0/3 |
|  | 50 | 3/3 | 0/3 |
|  | 20 | 3/3 | 0/3 |
|  | 10 | 0/3 | 0/3 |
| GBS | 1000 | 3/3 | 3/3 |
|  | 500 | 3/3 | 3/3 |
|  | 200 | 3/3 | 3/3 |
|  | 100 | 3/3 | 3/3 |
|  | 50 | 3/3 | 0/3 |
|  | 20 | 3/3 | 0/3 |
|  | 10 | 3/3 | 0/3 |
| GAS | 1000 | 3/3 | 3/3 |
|  | 500 | 3/3 | 3/3 |
|  | 200 | 3/3 | 3/3 |
|  | 100 | 3/3 | 3/3 |
|  | 50 | 3/3 | 0/3 |
|  | 20 | 3/3 | 0/3 |
|  | 10 | 0/3 | 0/3 |

**Note:** Simulated CSF samples were prepared at the indicated concentrations and tested in triplicate in three independent experiments. The data shown are from manual nucleic acid extraction. SPN, *Streptococcus pneumoniae*; GBS, *Streptococcus agalactiae*; GAS, *Streptococcus pyogenes*; mOTN-PCR, multiplex one-tube nested real-time polymerase chain reaction; real-time PCR, real-time polymerase chain reaction.

**Supplementary Table 5.** Reproducibility of mOTN-PCR and real-time PCR for detecting SPN, GBS, and GAS in simulated CSF samples after POCT extraction

| **Pathogen** | **Concentration (CFU/mL)** | **mOTN-PCR (Positive/Total)** | **real-time PCR (Positive/Total)** |
| --- | --- | --- | --- |
| SPN | 500 | 3/3 | 3/3 |
|  | 200 | 3/3 | 3/3 |
|  | 100 | 3/3 | 3/3 |
|  | 50 | 3/3 | 1/3 |
|  | 20 | 3/3 | 0/3 |
|  | 10 | 0/3 | 0/3 |
|  | 5 | 0/3 | 0/3 |
| GBS | 500 | 3/3 | 3/3 |
|  | 200 | 3/3 | 3/3 |
|  | 100 | 3/3 | 3/3 |
|  | 50 | 3/3 | 3/3 |
|  | 20 | 3/3 | 0/3 |
|  | 10 | 0/3 | 0/3 |
|  | 5 | 0/3 | 0/3 |
| GAS | 500 | 3/3 | 3/3 |
|  | 200 | 3/3 | 3/3 |
|  | 100 | 3/3 | 3/3 |
|  | 50 | 3/3 | 0/3 |
|  | 20 | 3/3 | 0/3 |
|  | 10 | 0/3 | 0/3 |
|  | 5 | 0/3 | 0/3 |

**Note:** Simulated CSF samples were prepared at the indicated concentrations and tested in triplicate in three independent experiments. The data shown are from fully automated nucleic acid extraction using the POCT instrument. SPN, *Streptococcus pneumoniae*; GBS, *Streptococcus agalactiae*; GAS, *Streptococcus pyogenes*; mOTN-PCR, multiplex one-tube nested real-time polymerase chain reaction; real-time PCR, real-time polymerase chain reaction; POCT, point-of-care testing.

**Supplementary Table 6.** Diagnostic performance of mOTN-PCR-POCT and real-time PCR compared to culture results in clinical samples (n = 109)

| **Method** | **Sensitivity% (n/N) [95% CI]** | **Specificity%**  **(n/N) [95% CI]** | **PPV% (n/N) [95% CI]** | **NPV% (n/N) [95% CI]** | **Overall Agreement% (n/N) [95% CI]** |
| --- | --- | --- | --- | --- | --- |
| **mOTN-PCR-POCT** | 100% (19/19)  [82.4-100%] | 100% (90/90) [96.0-100%] | 100% (19/19)  [82.4-100%] | 100% (90/90) [96.0-100%] | 100% (109/109) [96.7-100%] |
| **real-time PCR** | 84.2% (16/19) [60.4-96.6%] | 100% (90/90) [96.0-100%] | 100% (16/16)  [79.4-100%] | 96.8% (90/93) [90.3-99.3%] | 97.2% (106/109) [92.1-99.4%] |

**Note:** CI, confidence interval; PPV, positive predictive value; NPV, negative predictive value; mOTN-PCR, multiplex one-tube nested real-time polymerase chain reaction; real-time PCR, real-time polymerase chain reaction; POCT, point-of-care testing.
